# Inhibition of notch promotes liver metastasis

**DOI:** 10.18632/aging.100811

**Published:** 2015-09-20

**Authors:** Debarshi Banerjee, Angela Kadenhe-Chiweshe, Darrell J. Yamashiro

**Affiliations:** Irving Cancer Research Center, Columbia University Medical Center, New York, NY 10032, USA

**Keywords:** anti-Notch therapy, liver metastasis, sinusoidal endothelial cells, stellate cells

Classically known for its role in development and differentiation, an oncogenic role for Notch was first discovered for T-cell acute lymphoblastic leukemia [1], and later extended to a variety of tumors [2]. Notch signaling has been mechanistically linked to regulate tumor growth, angiogenesis and metastatic progression. Consequently, in the last decade we have witnessed multiple therapeutic approaches to target Notch, including γ-secretase inhibitors (GSIs), which block cleavage and activation of Notch receptors, soluble Notch decoy receptors, and antibodies to either Notch receptors or ligands [3]. However due to widespread function and highly pleotropic nature, Notch inhibition raises the possibility of unanticipated adverse effects in host tissues including gastrointestinal toxicity after GSI treatment or formation of vascular tumors after DLL4 ligand blockade [4, 5].

Our current work published in the journal *Cancer Research*, has raised one such concern about Notch inhibition therapy [6]. Inhibition of Notch with the soluble receptor Notch1 decoy (N1D), markedly increased liver metastatic burden, of neuroblastoma and breast cancer cells lines after intrarenally or intracardiac injection (spontaneous vs. experimental metastasis models). Additionally, pharmacologic inhibition of Notch with the GSI PF-03084014, while having no effect on primary tumor growth, significantly increased liver metastases. Interestingly, we did not observe in any of the experimental models, an increase in metastasis to other organs including spleen, kidney and bone marrow, suggesting that Notch blockade specifically promotes metastases to the liver.

Our studies determined that the increased liver metastases are a result of a direct effect of Notch inhibition on host liver, with the loss of tumor cell-intrinsic Notch1 signaling inconsequential. Tumor cells expressing N1D showed no change in prometastatic characteristics such as migration and invasion. Loss of Notch signaling in tumor cells by knocking down Notch1 also failed to promote liver metastases. However, intracardiac injection of tumor cells into immunodeficient (Rag2−/−, Il2rg−/−) Notch1 +/− mice, where host Notch signaling was deficient due to heterozygous deletion of *Notch,* demonstrated a marked increase in hepatic metastases, indicating that Notch1 signaling acts as metastatic suppressor in the liver microenvironment.

The liver microenvironment is composed of a variety of cell types, including sinusoidal endothelial cells (SECs), hepatic stellate cells (HSCs), and macrophage Kupffer cells, which express Notch receptors and ligands [7]. One striking characteristic of the liver metastases promoted by Notch blockade was their markedly increased vascularity. We observed a significant increase in the sprouting of hepatic SECs into early stage, micrometastases. Utilizing Notch decoy variants that are specific for the Notch ligands DLL and JAGGED, we determined that the increased sprouting was due to blockade of DLL on SECs. In the later stage, macrometastases, there was a marked increase in larger diameter vasculature that was α-smooth muscle actin (+), indicating activation and recruitment of HSCs into the vasculature. This was likely due to the loss of tumor derived JAG1, as indicated by blockade with a JAGGED specific decoy. Our data support a concept of Notch signaling maintaining the quiescence of SECs via DLL and HSCs via JAG1, with disruption of this quiescence promoting a prometastatic environment.

**Figure 1:**
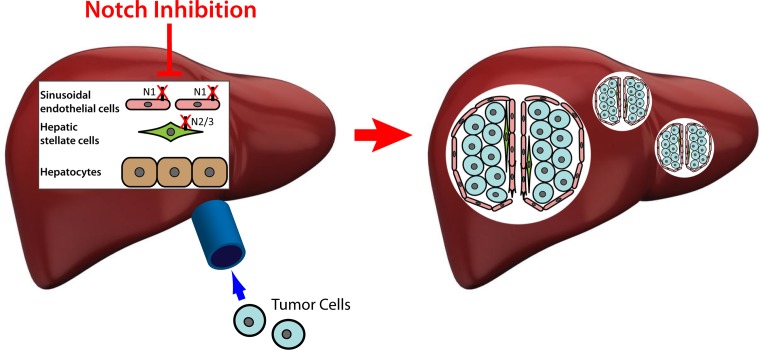
Notch inhibition activates sinusoidal endothelial cells and hepatic stellate cells to promote hepatic metastases.

Our study was primarily focused on Notch1, however, it would be interesting to investigate if other Notch receptors have similar roles since they are reported to regulate SEC and HSC function to maintain liver home-ostasis [7]. Given that metastasis progression is a multistep process it would also be interesting to investigate if Notch signaling regulates other steps including survival against host innate immunity. Nonetheless, our study shows that Notch inhibition results in pathological activation of liver stromal cells creating a host microenvironment favorable for metastases. Given that Notch components have emerged as potential therapeutic targets and Notch inhibitors are entering clinical trials these findings point out the potentially serious implication of Notch inhibition therapy.

## References

[1] Ellisen LW (1991). Cell.

[2] Ranganathan P (2011). Nat Rev Cancer.

[3] Takebe N (2015). Nat Rev Clin Oncol.

[4] van Es JH (2005). Nature.

[5] Yan M (2010). Nature.

[6] Banerjee D (2015). Cancer Res.

[7] Morell CM (2013). Clin Res Hepatol Gastroenterol.

